# Ribosome profiling of HEK293T cells overexpressing codon optimized coagulation factor IX

**DOI:** 10.12688/f1000research.22400.2

**Published:** 2020-09-21

**Authors:** Aikaterini Alexaki, Jacob Kames, Gaya K. Hettiarachchi, John C. Athey, Upendra K. Katneni, Ryan C. Hunt, Nobuko Hamasaki-Katagiri, David D. Holcomb, Michael DiCuccio, Haim Bar, Anton A. Komar, Chava Kimchi-Sarfaty

**Affiliations:** 1 Center for Biologics Evaluation and Research, Food and Drug Administration, USA, Silver Spring, MD, 20993, USA; 2 National Center of Biotechnology Information, National Institutes of Health, USA, Bethesda, MD, 20892, USA; 3 Department of Statistics, University of Connecticut, Storrs, CT, 06269, USA; 4 Center for Gene Regulation in Health and Disease, Cleveland State University, Cleveland, OH, 44115, USA

**Keywords:** Ribosome profiling, codon optimization, Ribo-seq, RNA-seq, translation kinetics, codon usage, codon pair usage, protein therapeutics

## Abstract

Ribosome profiling provides the opportunity to evaluate translation kinetics at codon level resolution. Here, we describe ribosome profiling data, generated from two HEK293T cell lines. The ribosome profiling data are composed of Ribo-seq (mRNA sequencing data from ribosome protected fragments) and RNA-seq data (total RNA sequencing). The two HEK293T cell lines each express a version of the *F9* gene, both of which are translated into identical proteins in terms of their amino acid sequences. However, these *F9 *genes vary drastically in their codon usage and predicted mRNA structure. We also provide the pipeline that we used to analyze the data. Further analyzing this dataset holds great potential as it can be used i) to unveil insights into the composition and regulation of the transcriptome, ii) for comparison with other ribosome profiling datasets, iii) to measure the rate of protein synthesis across the proteome and identify differences in elongation rates, iv) to discover previously unidentified translation of peptides, v) to explore the effects of codon usage or codon context in translational kinetics and vi) to investigate cotranslational folding. Importantly, a unique feature of this dataset, compared to other available ribosome profiling data, is the presence of the *F9* gene in two very distinct coding sequences.

## Introduction

The ribosome profiling (footprinting) technique has only been around for a decade ^1^ but has already contributed tremendously to our understanding of translation efficiency and kinetics. Initially developed to systematically monitor protein translation in yeast ^1^, it has since been adapted to work in a range of organisms ^2, 3^ and to tackle a variety of questions. Ribosome profiling data typically consist of a set of sequences of ribosome protected fragments (RPF), designated as Ribo-seq data, which is accompanied by sequences from total RNA (RNA-seq). The availability of Ribo-seq and RNA-seq data from the same sample provides a treasure trove of information, enabling quantitative study of translation efficiency, rate and kinetics of every mRNA sequence in the pool ^4^. Given that these sequences cover the entire transcriptome, and also include tRNA and rRNA, typically only a fraction of the data is presented and constructively used, within its initial publication. Further analyses, and comparisons of different ribosome profiling datasets can yield significant new information.

We recently conducted a ribosome profiling study to examine the translation kinetics of blood coagulation factor IX ^5^, a protein with great pharmaceutical interest. Two human embryonic kidney 293T (HEK293T) cell lines were lentivirally transduced, one with the wild type (WT) version of the gene and one with a codon optimized (CO) *F9* ^5^. Codon optimization is a widely used technique that aims at increasing the protein expression levels by replacing multiple codons within a coding sequence with synonymous ones. In doing so, the amino acid sequence of the protein remains unaltered, therefore these changes were assumed to be inconsequential for the structure and function of the protein. However, this is not always true; through our ribosome profiling study, we described that these synonymous changes drastically altered translational kinetics and led to protein conformational changes ^5^.

The translational kinetics of the *F9* variants, along with the control genes, *GAPDH* and *ACTB*, were analyzed in detail in the original publication ^5^. Similarly, any other gene of interest can be investigated in this dataset in terms of their rate of synthesis and translational kinetics; genes in the entire transcriptome can be compared to each other. Since there are several other HEK293T ribosomal profiling datasets available, these could be used to examine the reproducibility of the results ^6^. Furthermore, by looking into ribosome profiling datasets from other cell types, such as other human cells ^7^ and/or across species, it would be valuable to examine whether a given gene maintains the same translation kinetics or if there are significant differences that could reflect on the conformation of the protein. Clearly, since a rather large inter-experiment variation is expected, the accumulation of several ribosome profiling databases would be very useful for this type of analysis.

Innovative computational approaches of analyzing ribosome profiling data have led to the identification of novel CDSs that lead to the production of previously unidentified peptides and variants of known proteins ^8^. Such coding sequences may be found in what is typically designated as untranslated regions (UTRs) of the mRNA, particularly the 5’UTRs, and may originate from non-AUG start sites ^9– 11^. However, such approaches have not been applied yet to this dataset and it would be intriguing to see if they could lead to new discoveries ^12^. Importantly, since the genome of the HEK293T used to generate this dataset contains part of lentiviral vector and the cytomegalovirus (CMV) promoter to drive expression of *F9*, it would be interesting to examine whether any part of this sequence is actively translated. These analyses may be particularly insightful in studies of immunogenicity.

Further analysis of this dataset will help elucidate the effect of codon usage, codon context and possible other factors in translational kinetics. By looking at the global rate in which each codon is translated, and examining adjacent sequences on a transcriptome level, it may be possible to predict translational kinetics of recombinant genes and to make inferences on whether cotranslational folding may be affected. This may be particularly important in gene therapy applications where the cell type expressing the gene of interest may be different from the naturally expressing cells, e.g. expression of coagulation factor VIII from hepatocytes in gene therapy. A recent study in yeast ^13^ showed promising results in this direction; however, increasing availability of ribosome profiling datasets from other cell types will allow further comparisons. A unique feature of this dataset that may be pivotal in these types of studies is the presence of *F9* in two genes with very different codon usage.

## Materials and methods

### Plasmid/vector construction

WT (RefSeq NM_000133.3) and CO (accessible at https://github.com/FDA/Ribosome-Profiling F9_opt1_construct_100bpUTRs.fasta) ^14^ *F9* ORFs were sub-cloned into pcDNA3.1/V5-His-TOPO (Invitrogen/Life Technologies) according to manufacturer’s instructions to generate pcDNA3.1- *F9*-V5-His plasmids. Each fusion construct (WT *F9*-V5-His and CO *F9*-V5-His) was sub-cloned into a lentiviral vector pTK642 (gift from Dr. Kafri, University of North Carolina at Chapel Hill) at the Pacl/Sfil site.

### Cell cultures and lentiviral transduction

Human embryonic kidney cells (HEK293T; ATCC) were grown in Dulbecco’s Modified Eagle Medium (Quality Biological, Inc) with 1% L-glutamine (Quality Biological), 1% penicillin- streptomycin (Hyclone) and 10% fetal bovine serum (Quality Biological) at 37°C in 5% CO _2_. HEK293T cells stably expressing WT or CO FIX were established following transduction with lentiviral vectors, as previously described ^15^.

An equivalent number of cells were plated in T-flasks and supplemented with 10 ng/ml of Vitamin K3, one day prior to all experiments. The culture medium was replaced with Opti-MEM Reduced Serum Medium (Life Technologies) at approximately 80–90% cell confluency and cells were harvested after an additional 24 hours of incubation. 

### Ribosome profiling

Ribosome profiling was conducted as described previously ^7^ using the Illumina TruSeq Ribo Profile (Mammalian) Kit according to manufacturer’s instructions with modifications in harvest, RNA isolation/purification (isopropanol isolation used to improve the yield) and ribosome protected fragments size selection (~20–32 nt). During harvest, media was carefully removed, and cells were immediately flash-frozen. All equipment used from hence forth was pre-chilled. Cells were quickly scraped into 1 ml of ice-cold lysis buffer (5X Mammalian Polysome Buffer, 10% Triton-X100, 100 mM DTT, DNase I, Nuclease-free water) and homogenized on ice by passing through a 26G needle 10 times. Lysate was then spun at 4°C for 10 minutes at 20,000 × g. Supernatant was aliquoted into cryovials and immediately frozen in liquid nitrogen for future use. Samples were sequenced using Illumina HiSeq 2500.

**Figure 1.**
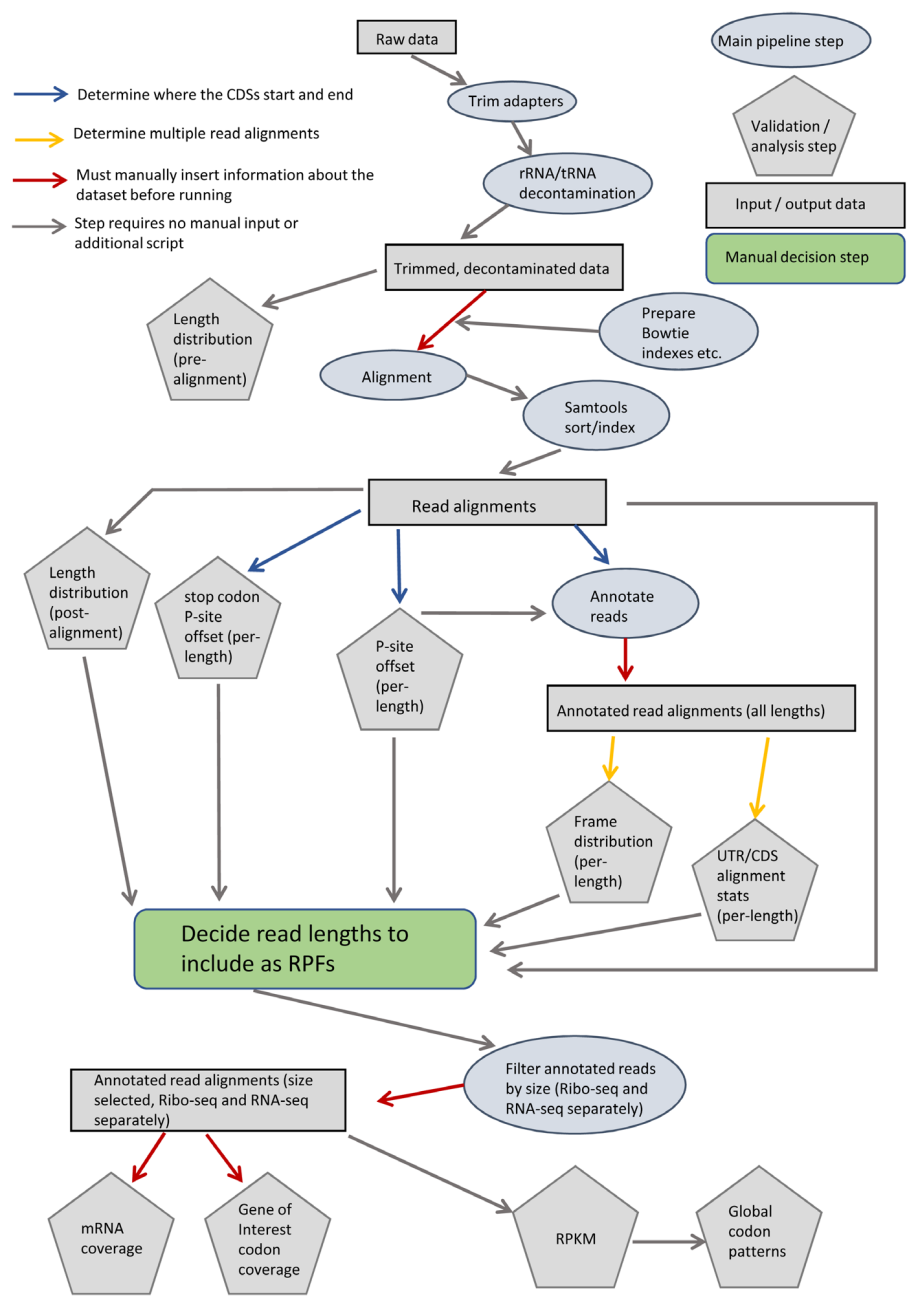
Flowchart of ribosome profiling data analysis pipeline. Colored arrows indicate steps that first require execution of utility script (blue and yellow) or require manual input by the user (red). Pipeline steps are represented as ovals (main step) or pentagons (validation / analysis step). Rectangles represent input / output data. UTR: untranslated region, CDS: coding sequence, RPF: ribosome protected fragments, RPKM: reads per kilobase of transcript per million mapped reads.

The complete ribosome profiling pipeline analysis is described in Figure 1: Sequencing data were pre-processed and aligned as described by Alexaki *et al*. ^5^ as well as the step by step guide found in the README.txt accessible on GitHub.

**Figure 2.**
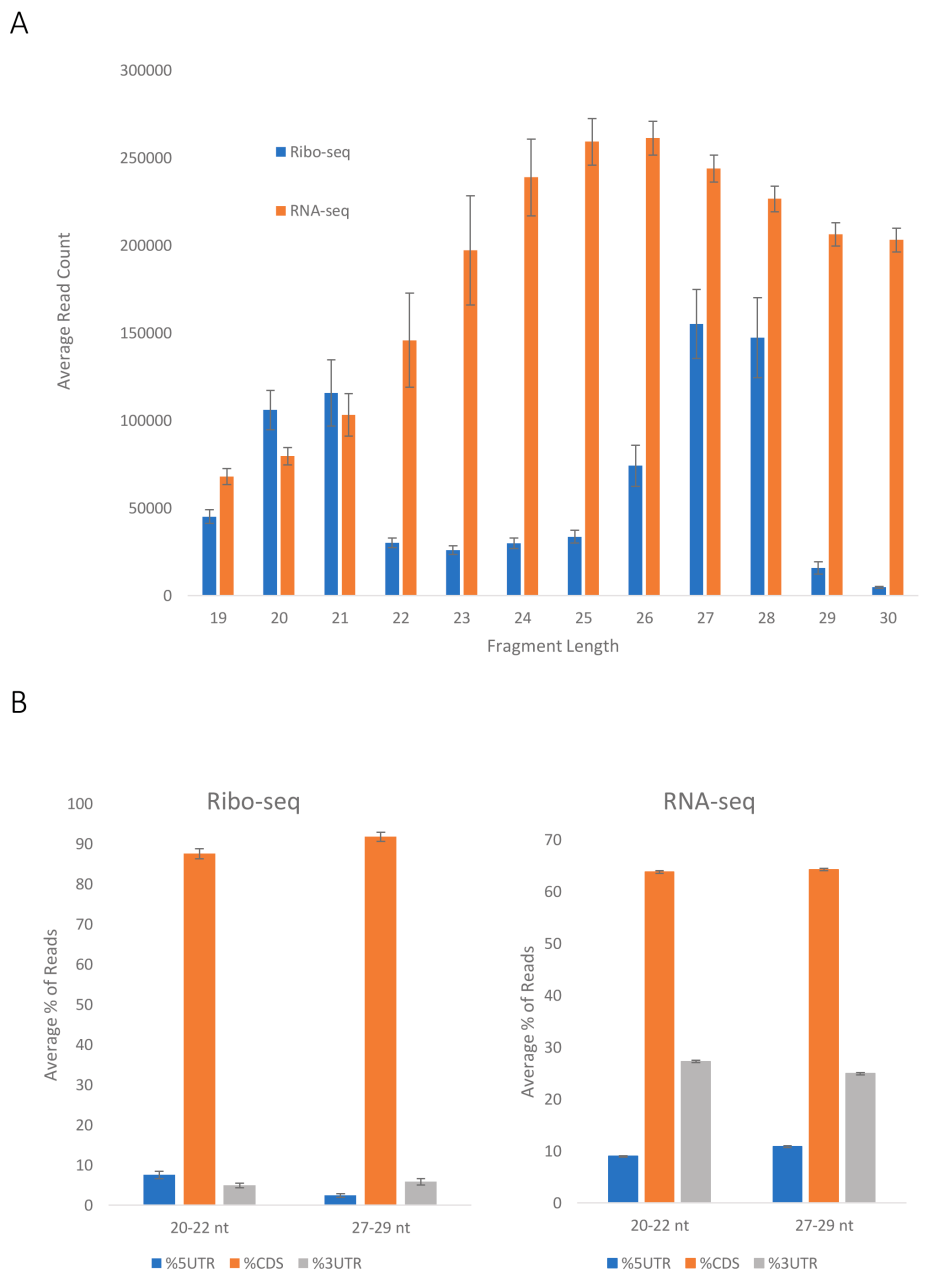
Ribo-Seq and RNA-Seq data distribution. ( **a**) Fragment size distribution of Ribo-seq and RNA-seq reads. The average of 6 experiments (3 WT and 3 CO *F9*) was plotted, s.e.m. are shown. ( **b**) Distribution of Ribo-seq (left) and RNA-seq (right) reads in mRNA coding regions (CDSs) and untranslated (5’UTR and 3’UTR) regions. The average of 6 experiments (3 WT and 3 CO *F9*) was plotted, s.e.m. are shown.

**Figure 3.**
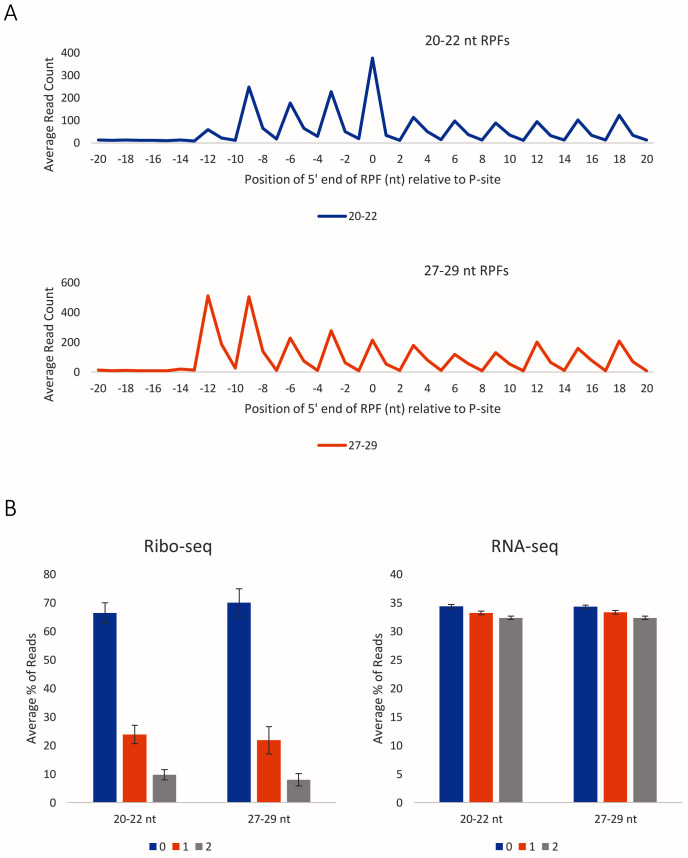
Triplet periodicity of Ribo-Seq data. ( **a**) Profiles of the 5′ end positions of all 20–22 nt (top) and 27–29 nt (bottom) fragments relative to the start codon of their genes. The average of 6 experiments (3 WT and 3 CO *F9*) was plotted. ( **b**) Positions of 20–22 nt and 27–29 nt fragments relative to the reading frame of the Ribo-seq (left) and RNA-seq (right) reads. The average of 6 experiments (3 WT and 3 CO *F9*) was plotted, s.e.m. are shown.

RPF sequences were analyzed based on fragment length ( Figure 2a), alignment distribution between coding sequences (CDSs) and 5’- and 3’-UTRs ( Figure 2b), triplet periodicity ( Figure 3a) and reading frame ( Figure 3b). RPF fragments 20–22 nt and 27–29 nt in length were used for further analysis with a P-site offset of 12 nucleotides from the 5’ end of the fragment. Pearson and Spearman correlations were used to evaluate the reproducibility between replicates using a common subset of moderately to highly expressed genes (reads per kilobase of transcript per million mapped reads, RPKM _CDS_ ≥10) and considering reads with the ribosome A site annotated at least 20 nt downstream of the coding sequence start codon ( Table 1). Both Pearson and Spearman coefficients show strong correlation between experimental replicates.

**Table 1.** Pearson and Spearman correlations between pairs of experiments. RPKM of each gene in the Ribo-seq and RNA-seq datasets were calculated, considering reads with the ribosome A site annotated at least 20 nt downstream of the start codon. A comparison between each pair of experiments within the 3 replicates was performed

Experiment	Ribo-Seq (Pearson)	RNA-Seq (Pearson)	Ribo-Seq (Spearman)	RNA-Seq (Spearman)
WT1-WT2	0.9973	0.9958	0.9426	0.9775
WT2-WT3	0.9972	0.9976	0.9513	0.9785
WT1-WT3	0.9962	0.9917	0.9384	0.9774
CO1-CO2	0.9908	0.9979	0.9314	0.9755
CO2-CO3	0.9927	0.998	0.9282	0.9771
CO1-CO3	0.994	0.9979	0.9428	0.9771

### Dataset validation

The quality of the sequencing files is presented in Table 2. A pipeline was created to process the data ( Figure 1). A number of steps allow for validation of the data and confirmation of their quality. The fragment length distributions for the whole genome were plotted, indicating that the vast majority of the fragments from the Ribo-seq data are either 20–21 or 27–28 nucleotides in length ( Figure 2a), and as expected the RNA-seq data have a more flat distribution. The distribution of the Ribo-seq data in the UTRs and CDSs of the mRNA was also plotted. As expected, most of the sequences aligned within the CDSs ( Figure 2b), while a smaller fraction of the RNA-seq data aligned with the CDSs. It should be noted that as the 3’ UTR, and 5’ UTR are typically shorter in length than the CDSs, it is not surprising that about 60% of the RNA-seq data align with the CDSs ( Figure 2b). In addition, Ribo-seq data exhibit periodicity, characteristic of the RPFs ( Figure 3a and Figure 3b), which is not observed in the RNA-seq data ( Figure 3b). In accordance with previously published data ^16^, we can infer that the 5′-most peaks in ( Figure 3a) represent ribosomes with the start codon in the P site and the second codon in the A site, for both large and short fragments. Very tight correlation between the experiments, both for Ribo-seq and RNA-seq data, supports the reproducibility of the results ( Table 1).

**Table 2.** Quality data of sequencing files. Sample ID, index, yield, number of clusters, percent Q30 and above and mean Q score for all sequencing experiments.

Sample	Index	Yield (Mbp)	#Cluster	%Q30	Mean Q
1R	CAGATC	1,669	13,355,848	66.82	25.8
1T	ATCACG	1,821	14,566,314	79.59	30.33
2R	ACTTGA	1,681	13,451,867	71.56	27.47
2T	CGATGT	1,867	14,932,652	78.55	29.96
3R	GATCAG	1,512	12,092,292	71.18	27.36
3T	TTAGGC	1,653	13,227,113	79.74	30.37
4R	TAGCTT	1,825	14,600,572	68.43	26.38
4T	TGACCA	1,731	13,848,340	79.75	30.38
5R	GGCTAC	1,537	12,292,279	67.63	26.08
5T	ACAGTG	1,754	14,033,677	80.22	30.55
6R	CTTGTA	1,818	14,541,142	68.64	26.44
6T	GCCAAT	1,662	13,296,276	78.6	29.97

### Data records

Sequencing for 3 replicates of RNA-seq and Ribo-seq of HEK293T cells stably expressing WT and CO FIX was performed by Eurofins Genomics (Louisville, KY, USA), resulting in 12 raw data files (3 WT and 3 CO *F9* for both Ribo-seq and RNA-seq) in FASTQ format. Raw data are accessible at the NCBI Sequence Read Archive (SRA) under BioProject accession PRJNA591214. File names, SRA accession numbers (experiment and sample) and descriptions of data are summarized below in Table 3.

**Table 3.** Description of data deposited in SRA. Filenames, SRA experiment accession, SRA sample accession and brief description of the 12 Ribo-seq and RNA-seq FASTQ files. All data files are accessible from SRA BioProject accession PRJNA591214. Data files represent three replicates of each condition (WT *F9* Ribo-seq, WT *F9* RNA-seq, CO *F9* Ribo-seq and CO *F9* RNA-seq).

Filename	SRA Experiment	SRA Sample	Description
1R_CAGATC_L002_R1_001.fastq.gz	SRX7201733	SAMN13354200	WT F9 RIBO-SEQ 1
1T_ATCACG_L002_R1_001.fastq.gz	SRX7201734	SAMN13354201	WT F9 mRNA-SEQ 1
2R_ACTTGA_L002_R1_001.fastq.gz	SRX7201737	SAMN13354202	WT F9 RIBO-SEQ 2
2T_CGATGT_L002_R1_001.fastq.gz	SRX7201738	SAMN13354203	WT F9 mRNA-SEQ 2
3R_GATCAG_L002_R1_001.fastq.gz	SRX7201739	SAMN13354204	WT F9 RIBO-SEQ 3
3T_TTAGGC_L002_R1_001.fastq.gz	SRX7201740	SAMN13354205	WT F9 mRNA-SEQ 3
4R_TAGCTT_L002_R1_001.fastq.gz	SRX7201741	SAMN13354206	CO F9 RIBO-SEQ 1
4T_TGACCA_L002_R1_001.fastq.gz	SRX7201742	SAMN13354207	CO F9 mRNA-SEQ 1
5R_GGCTAC_L002_R1_001.fastq.gz	SRX7201743	SAMN13354208	CO F9 RIBO-SEQ 2
5T_ACAGTG_L002_R1_001.fastq.gz	SRX7201744	SAMN13354209	CO F9 mRNA-SEQ 2
6R_CTTGTA_L002_R1_001.fastq.gz	SRX7201735	SAMN13354210	CO F9 RIBO-SEQ 3
6T_GCCAAT_L002_R1_001.fastq.gz	SRX7201736	SAMN13354211	CO F9 mRNA-SEQ 3

### Usage notes

The custom ribosome profiling analysis pipeline has been deposited in GitHub in the FDA/Ribosome-Profiling directory ^14^. Raw data files may be accessed from SRA and downloaded to the ‘./Ribosome_profiling/Raw_data/X/’ folder. In our descriptions and instructions, ‘X’ is replaced with ‘S12’, but the user may choose any designation they prefer. Detailed instructions for running the data analysis pipeline are included in the ‘README.txt’ file.

Execution of the pipeline requires the following tools (version tested) be installed on the user’s system: Python (3.7.6) ( https://www.python.org) (Python Software Foundation, Wilmington, DE, USA) and modules pysam (0.15.3) ( https://github.com/pysam-developers/pysam) and biopython (1.77) ( https://biopython.org/), GFF Utilities (gffread v0.12.1) ( http://ccb.jhu.edu/software/stringtie/gff.shtml) (Johns Hopkins University, Baltimore, MD, USA), Bowtie (1.0.0) ( http://bowtie-bio.sourceforge.net/index.shtml) (Johns Hopkins University, Baltimore, MD, USA), HISAT2 (2.1.0) ( https://ccb.jhu.edu/software/hisat2/manual.shtml) (Johns Hopkins University, Baltimore, MD, USA), FASTX-Toolkit (0.0.14) ( http://hannonlab.cshl.edu/fastx_toolkit/commandline.html) (Cold Spring Harbor Laboratory, Cold Spring Harbor, NY, USA), Samtools (1.7 using htslib 1.7) ( http://www.htslib.org/) (Genome Research Limited, Hinxton, Cambridgeshire, UK).

## Data availability

NCBI BioProject: Ribosome profiling of HEK-293T cells stably expressing wild-type and codon-optimized coagulation factor IX. Accession number PRJNA591214; https://identifiers.org/NCBI/bioproject:PRJNA591214.

This project collates the raw data, held at the NCBI Sequence Read Archive (SRA).

## Software availability

**The pipeline, including the code used to process the presented dataset and instructions for use, is available:** https://github.com/FDA/Ribosome-Profiling

**Archived pipeline at time of publication:** https://doi.org/10.5281/zenodo.3678709 ^14^.

**License:** MIT License.
