# Supplementary material for: Ribosome profiling of HEK293T cells overexpressing codon optimized coagulation factor IX
Source: F1000Res. 2020 Sep 21;9:174. Originally published 2020 Mar 10. [Version 2] doi: 10.12688/f1000research.22400.2 (PMC7509596; doi:10.12688/f1000research.22400.2)
Supplement: Peer review history

# Peer review: Reviewer response for version 2

**DOI:** 10.5256/f1000research.29425.r71732

**Published:** 2020-10-07

**Authors:** James Collawn

**Affiliations:** 1 Department of Cell, Developmental and Integrative Biology, University of Alabama at Birmingham, Birmingham, AL, USA

I am fine with the responses of the authors. I have no further concerns.

Are sufficient details of methods and materials provided to allow replication by others?

Yes

Is the rationale for creating the dataset(s) clearly described?

Yes

Are the datasets clearly presented in a useable and accessible format?

Yes

Are the protocols appropriate and is the work technically sound?

Yes

Reviewer Expertise:

NA

I confirm that I have read this submission and believe that I have an appropriate level of expertise to confirm that it is of an acceptable scientific standard.

# Peer review: Reviewer response for version 2

**DOI:** 10.5256/f1000research.29425.r71730

**Published:** 2020-09-28

**Authors:** Stefano Biffo

**Affiliations:** 1 INGM, National Institute of Molecular Genetics, “Fondazione Romeo ed Enrica Invernizzi”, Milan, Italy

Are sufficient details of methods and materials provided to allow replication by others?

Yes

Is the rationale for creating the dataset(s) clearly described?

Yes

Are the datasets clearly presented in a useable and accessible format?

No

Are the protocols appropriate and is the work technically sound?

Yes

Reviewer Expertise:

NA

I confirm that I have read this submission and believe that I have an appropriate level of expertise to confirm that it is of an acceptable scientific standard.

# Peer review: Reviewer response for version 2

**DOI:** 10.5256/f1000research.29425.r71731

**Published:** 2020-09-22

**Authors:** Jordan Berg

**Affiliations:** 1 Department of Biochemistry, University of Utah, Salt Lake City, UT, USA

The changes look good. My only final recommendation would be to version the pipeline again with the updates and make sure the updated repository is archived in Zenodo (it looks like it only has the version from Feb 2020 archived).

Are sufficient details of methods and materials provided to allow replication by others?

Partly

Is the rationale for creating the dataset(s) clearly described?

Yes

Are the datasets clearly presented in a useable and accessible format?

Partly

Are the protocols appropriate and is the work technically sound?

Partly

Reviewer Expertise:

Ribosome profiling library creation and data analysis.

I confirm that I have read this submission and believe that I have an appropriate level of expertise to confirm that it is of an acceptable scientific standard.

# Peer review: Reviewer response for version 1

**DOI:** 10.5256/f1000research.24715.r65798

**Published:** 2020-07-14

**Authors:** James Collawn, Rafal Bartoszewski

**Affiliations:** 1 Department of Biology and Pharmaceutical Botany, Medical University of Gdansk, Gdansk, Poland; 2 Department of Cell, Developmental and Integrative Biology, University of Alabama at Birmingham, Birmingham, AL, USA

This study by Dr. Kimchi-Sarfaty examines ribosome profiling to follow the translational kinetics of factor IX (F9) and is a follow up of a previous study of theirs (DOI: 10.1038/s41598-019-51984-2) ^1^. They compare two HEK293T cell lines with one expressing a WT F9 gene and another with a codon-optimized F9 gene. In their previous study, they demonstrated the importance of this type of analysis by illustrating that these two cell lines had different translational kinetics and furthermore, different effects on protein conformation. In this study, they compared 3 replicates of the RNA-seq and Ribo-seq of the two cell lines. The correlations between the pairs of experiments seemed quite good and we believe this is an important type of study, however, we do have two concerns. First of all, the housekeeping control genes they utilize are GAPDH and ACTB, both of which are translated on free ribosomes in the cytosol. F9, however, is a secreted protein that is translated on ER ribosomes. Therefore, a rationale for the appropriateness of the two controls needs to be discussed. Secondly, they used a Box-Cox variance-stabilizing transformation for raw data followed by a Kolmogorov-Smirnov test which tests for probability distribution functions but is a less selective normality test in our view. Why not utilize Saphiro-Wilk ( https://doi.org/10.1093/biomet/52.3-4.591 )[ref-2] or Arednson-Darling tests ( doi: 10.2307/2281537) ^3^? Some more detail here about the rationale for the statistical analyses is therefore warranted and whether additional controls and samples would have any effect on the outcomes of the studies should be discussed.

Are sufficient details of methods and materials provided to allow replication by others?

Yes

Is the rationale for creating the dataset(s) clearly described?

Yes

Are the datasets clearly presented in a useable and accessible format?

Yes

Are the protocols appropriate and is the work technically sound?

Yes

Reviewer Expertise:

Protein trafficking, protein expression and folding (Collawn); mRNA structure, translational expression, and silent polymorphisms (Bartoszewski).

We confirm that we have read this submission and believe that we have an appropriate level of expertise to confirm that it is of an acceptable scientific standard, however we have significant reservations, as outlined above.

## References

[1] : Effects of codon optimization on coagulation factor IX translation and structure: Implications for protein and gene therapies. *Scientific Reports*.2019;9(1) : 10.1038/s41598-019-51984-2 10.1038/s41598-019-51984-2 31664102PMC6820528

[2] : An analysis of variance test for normality (complete samples). *Biometrika*.1965;52(3-4) : 10.1093/biomet/52.3-4.591 591-611 10.1093/biomet/52.3-4.591

[3] : A Test of Goodness of Fit. *Journal of the American Statistical Association*.1954;49(268) : 10.2307/2281537 10.2307/2281537

# Response

**Published:** 2020-09-04

**Authors:** Chava Kimchi-Sarfaty

**Affiliations:** Division of Hematology Research and Review, Food and Drug Administration, USA

Thank you very much for raising this interesting question. Although they are not secreted proteins as F9, ACTB and GAPDH were chosen as they have a relatively high level of expression and therefore generate sufficient sequencing reads to lead to a well resolved ribosome profile. Moreover, these two genes’ expression is stable and not changing due to the transfection or cell culture growth. It is not our intention to make any conclusions regarding the translation kinetics of secreted versus non-secreted proteins. Rather, the control genes are compared to themselves between experimental groups (cells expressing WT F9 and cells expressing CO F9). In this context, we believe that ACTB and GAPDH are appropriate to use as control genes.

 Regarding the statistical method, we want to point out that the Kolmogorov-Smirnov method is considered more conservative than the methods mentioned by the reviewer. Therefore, we feel that by using it we are able to detect a difference between two cumulative curves, then we should be more confident that the difference is real. Second, our aim is not to test for normality, but rather to compare two distributions. The main aims of the normalization step via the Box-Cox transformation is to stabilize the variance (of the originally skewed distribution), and to put the distributions on a comparable scale, so that we can compare the cumulative plots.

# Peer review: Reviewer response for version 1

**DOI:** 10.5256/f1000research.24715.r65801

**Published:** 2020-07-13

**Authors:** Jordan Berg

**Affiliations:** 1 Department of Biochemistry, University of Utah, Salt Lake City, UT, USA

The provided dataset provides the opportunity for more in-depth study of two versions of the *F9* gene with identical protein amino acid sequence but different nucleotide coding sequences, thus allowing for an exploration of the consequences of differential codon usage. The dataset itself is of biological and pharmacological interest and offers a valuable data contribution. More specific comments and questions regarding key points of the data note are provided below.

**Introduction:**

- At face value, this seems like a re-publishing of a dataset that has already been described in the literature (albeit with some additional detail and the pipeline). Were any data added or modified between the original publication and this data note? It is not clear why this dataset was not published with the original study describing these data ( https://doi.org/10.1038/s41598-019-51984-2) ^1^.
- If part of the intended contribution of this data note is the pipeline, it would be helpful to at least provide a bash script that ties together the different scripts and flexibly accepts input FASTQ files. Currently, with all the hard-coding present, it makes it difficult to reuse these scripts, especially when input references or sequence files vary from those used when analyzing this particular dataset.

**Methods:**

- A Pearson's correlation assumes the data are normally distributed. As sequencing data follows a negative binomial distribution, the use of a Spearman rank-order coefficient would be more appropriate. It might be helpful to clarify that this is for comparing biological replicates.
- Is there a reason the A-site offset is set at a strict 15 nt? Recent methods, such as those found in the RiboWaltz ( https://doi.org/10.1371/journal.pcbi.1006169) ^2^ package allow for a more optimized P-site offset determination that could probably be applied to A-site offset determination. On that note, is there a downstream reason you are interested in calculating the offset of the A-site? It looks like P-sites were used when calculating periodicity in the figures and scripts and thus it is not clear why the A-site offset is mentioned.
- The pipeline uses deprecated software (TopHat has been deprecated for nearly 5 years now) and should be updated to use a more accurate splice-aware option (such as STAR, HISAT2). Figures might need to be updated as appropriate. Is the installation of dependencies included in a script, or does the user need to handle that? For example, BioPython and pysam are included as a dependency in the python scripts, but I don't see them listed in the manuscript.

**GitHub Documentation:**

- To aid in readability, some formatting updating (new lines in example code) would be helpful. Currently, there are several commands on the same line as a comment, making the commands and comments difficult to read. Using markdown syntax to display the code sections would aid in readability as well.
- Explicitly state the gene annotation used for the preparation of this dataset. Comprehensive, basic gene, etc? Same for the GFF3 file. It is not clear from the documentation itself what versions were used and would be nice from an archiving/reproducibility stand-point. The same goes for listing software versions used for processing the dataset as presented in the paper.

**Code:**

- I could not get the *trim-adapters.py *script to work using the deposited data. I had empty trimmed files output and no error information was displayed to aid in debugging.
- As far as functionality, all of the indexing scripts appeared to function, but I was unable to test past the trim-adapters.py script due to the issue mentioned above.

Are sufficient details of methods and materials provided to allow replication by others?

Partly

Is the rationale for creating the dataset(s) clearly described?

Yes

Are the datasets clearly presented in a useable and accessible format?

Partly

Are the protocols appropriate and is the work technically sound?

Partly

Reviewer Expertise:

Ribosome profiling library creation and data analysis.

I confirm that I have read this submission and believe that I have an appropriate level of expertise to confirm that it is of an acceptable scientific standard, however I have significant reservations, as outlined above.

## References

[1] : Effects of codon optimization on coagulation factor IX translation and structure: Implications for protein and gene therapies. *Scientific Reports*.2019;9(1) : 10.1038/s41598-019-51984-2 10.1038/s41598-019-51984-2 31664102PMC6820528

[2] : riboWaltz: Optimization of ribosome P-site positioning in ribosome profiling data. *PLoS Comput Biol*.14(8) : 10.1371/journal.pcbi.1006169 e1006169 10.1371/journal.pcbi.1006169 30102689PMC6112680

# Response

**Published:** 2020-09-04

**Authors:** Chava Kimchi-Sarfaty

**Affiliations:** Division of Hematology Research and Review, Food and Drug Administration, USA

Thank you very much for your thorough review. Below we provide a point by point response.

- At face value, this seems like a re-publishing of a dataset that has already been described in the literature (albeit with some additional detail and the pipeline). Were any data added or modified between the original publication and this data note? It is not clear why this dataset was not published with the original study describing these data ( https://doi.org/10.1038/s41598-019-51984-2) ^1^.

*Regrettably, due to space restrictions we did not have the opportunity to publish the pipeline and the raw data with a detailed description in the original publication, which focused on the comparison of the wild type and codon optimized coagulation factor 9. We strongly believe that the raw ribosome profiling data can be of value to the scientific community and should be public with sufficient description to make it accessible. Similarly, we hope that the pipeline could be of use to researchers, and we note that despite the multitude of ribosome profiling papers that have been published so far, the software used in the analysis is not always readily accessible.*

  

- If part of the intended contribution of this data note is the pipeline, it would be helpful to at least provide a bash script that ties together the different scripts and flexibly accepts input FASTQ files. Currently, with all the hard-coding present, it makes it difficult to reuse these scripts, especially when input references or sequence files vary from those used when analyzing this particular dataset.

*We appreciate the suggestion and have made changes accordingly. The pipeline has been modified to be more user friendly, and updates have been made as described in the response to Reviewer 1.*

**Methods:**

- A Pearson's correlation assumes the data are normally distributed. As sequencing data follows a negative binomial distribution, the use of a Spearman rank-order coefficient would be more appropriate. It might be helpful to clarify that this is for comparing biological replicates.

*Thank you for pointing this out. We have added a Spearman rank-order coefficient to Table 1 for each of the dataset comparisons. The text describing Table 1 has been updated.*

- Is there a reason the A-site offset is set at a strict 15 nt? Recent methods, such as those found in the RiboWaltz ( https://doi.org/10.1371/journal.pcbi.1006169) ^2^ package allow for a more optimized P-site offset determination that could probably be applied to A-site offset determination. On that note, is there a downstream reason you are interested in calculating the offset of the A-site? It looks like P-sites were used when calculating periodicity in the figures and scripts and thus it is not clear why the A-site offset is mentioned.

*Thank you for pointing out the lack of clarity regarding the A- and P-site offset. The A-site offset at 15 nt is equivalent to a P-site offset at 12 nt. We made changes to consistently refer to the P-site offset.  We agree that when the translation frame is undetermined a hard 12 nt P-site offset can lead to inaccuracies, and an optimized method for P-site determination is paramount. However, our analysis did not incorporate unannotated open reading frames (ORFs), non-conventional translation initiation sites and we did not attempt to reveal novel translated regions. As a result, the triplet periodicity in our dataset is unambiguous and the P-site offset can be determined at 12 nt. For ribosome-protected fragments that aligned in reading frame one or two, a +/- 1 offset was additionally added to properly annotate the codons within each site of the ribosome.*

- The pipeline uses deprecated software (TopHat has been deprecated for nearly 5 years now) and should be updated to use a more accurate splice-aware option (such as STAR, HISAT2). Figures might need to be updated as appropriate. Is the installation of dependencies included in a script, or does the user need to handle that? For example, BioPython and pysam are included as a dependency in the python scripts, but I don't see them listed in the manuscript.

*We agree with your concern. When we started developing our pipeline, TopHat was the software of choice and we retained it to allow duplication of the data in our published paper [1]. We have now updated the pipeline to use HISAT2; all the figures have been updated accordingly. The readme file and manuscript have been updated to reflect the complete list of software dependencies and their versions.*

**GitHub Documentation:**

- To aid in readability, some formatting updating (new lines in example code) would be helpful. Currently, there are several commands on the same line as a comment, making the commands and comments difficult to read. Using markdown syntax to display the code sections would aid in readability as well.

*We thank you very much for pointing this out. We have removed all unnecessary commands and lines of code that had been commented out. We believe this will aid in readability of the code.*

  

- Explicitly state the gene annotation used for the preparation of this dataset. Comprehensive, basic gene, etc? Same for the GFF3 file. It is not clear from the documentation itself what versions were used and would be nice from an archiving/reproducibility stand-point. The same goes for listing software versions used for processing the dataset as presented in the paper.

*We agree with your concern. We have updated the GitHub documentation to explicitly state which annotation and assembly versions were used in the in the analysis of this dataset.*

- I could not get the *trim-adapters.py *script to work using the deposited data. I had empty trimmed files output and no error information was displayed to aid in debugging.As far as functionality, all of the indexing scripts appeared to function, but I was unable to test past the trim-adapters.py script due to the issue mentioned above.

*Thank you very much for pointing this out. This is an interesting result. We have re-run the analysis pipeline using the new bash scripts and code updated to Python 3.7, and the adapter trimming step ran correctly. However, we have added an error message in the Trim_adapters.py script to notify the user if an error occurs to aid in the debugging process.*

  

- Are the protocols appropriate and is the work technically sound? Partly

*We have updated the alignment tool used in the pipeline to HISAT2 and the code used in the individual scripts to Python 3.7. We hope that these updates to the pipeline have improved the appropriateness of the protocol.*

  

- Are sufficient details of methods and materials provided to allow replication by others? Partly

*We have bundled all scripts in the pipeline into two shell scripts and there is now a warning message in the Trim_adapters.py script that notifies the user when it does not execute properly. We have also updated the dependency list to accurately reflect all additional software required as well as versions that were tested. We hope that these updates have made replication of the work easier for the user.*

  

- Are the datasets clearly presented in a useable and accessible format? Partly

*We hope that the updates to the pipeline described above have made the datasets more useable and accessible.*

 1             Alexaki, A. *et al.* Effects of codon optimization on coagulation factor IX translation and structure: Implications for protein and gene therapies. *Sci Rep* **9**, 15449, (2019).

# Peer review: Reviewer response for version 1

**DOI:** 10.5256/f1000research.24715.r64172

**Published:** 2020-06-05

**Authors:** Stefano Biffo, Riccardo Rossi

**Affiliations:** 1 INGM, National Institute of Molecular Genetics, “Fondazione Romeo ed Enrica Invernizzi”, Milan, Italy; 2 INGM, Milan, Italy

The report is certainly interesting and the possibility to access data relatively nice. However, in the present form there is not much advantage compared to a paper supplementary section and accession number.

 In the current pipeline one should install - as per explicit requirements - Python 2.7, Bowtie, Tophat, Samtools, which still requires bioinformatician work.

 Some programs are not used anymore. Python 2 has been disconnected since January. Tophat as mapping resource is not suggested even by the developers, since now there are better ones. In the present form the pipeline can clearly work. In order to facilitate the use of the pipeline the authors could have wrapped everything in a folder that could have been run by the readers more easily.

 The biological data seem very nice to me (Stefano Biffo with the help of a bioinformatician).

Are sufficient details of methods and materials provided to allow replication by others?

Yes

Is the rationale for creating the dataset(s) clearly described?

Yes

Are the datasets clearly presented in a useable and accessible format?

No

Are the protocols appropriate and is the work technically sound?

Yes

Reviewer Expertise:

Translational control of gene expression

We confirm that we have read this submission and believe that we have an appropriate level of expertise to confirm that it is of an acceptable scientific standard, however we have significant reservations, as outlined above.

# Response

**Published:** 2020-06-19

**Authors:** Chava Kimchi-Sarfaty

**Affiliations:** Division of Hematology Research and Review, Food and Drug Administration, USA

Dr. Biffo,

Thank you very much for the time you put into reviewing the article and for your suggestions. We would like to inform you that we are currently working on upgrading the pipeline as per your comments. We will be updating the code to Python 3, bundling individual Python scripts into a more easily executable shell script and replacing the current Tophat alignment with a more modern tool. We very much appreciate your comments and we will notify you when we have completed the upgrades.

Chava Kimchi-Sarfaty and Jacob Kames

# Response

**Published:** 2020-09-04

**Authors:** Chava Kimchi-Sarfaty

**Affiliations:** Division of Hematology Research and Review, Food and Drug Administration, USA

We appreciate the suggestion and have made changes accordingly. The Python scripts used in the code have been updated to Python 3.7. Furthermore, the alignment software used in the pipeline has been changed from TopHat to Hisat2. Finally, two bash scripts have been created to run the pipeline. The first, *build_hisat_index.sh*, carries out the commands to set up the reference index with the two F9 constructs used in this study. The second, *RP_analysis_pipeline.sh*, runs the remaining steps of the pipeline. Both are run from within the *Ribosome_profiling* directory and have the dataset defined within each script.

We hope the reviewer agrees that the current changes make the software more user friendly and thus usable and accessible.
